# Structural Organization of the Presynaptic Density at Identified Synapses in the Locust Central Nervous System

**DOI:** 10.1002/cne.22744

**Published:** 2011-08-08

**Authors:** Gerd Leitinger, Sergej Masich, Josef Neumüller, Maria Anna Pabst, Margit Pavelka, F Claire Rind, Oleg Shupliakov, Peter J Simmons, Dagmar Kolb

**Affiliations:** 1 Institute of Cell Biology, Histology and Embryology, Center for Molecular Medicine (ZMM), Medical University of GrazAustria; 2 Core Facility Ultrastructure Analysis, Center for Medical Research (ZMF), Medical University of GrazAustria; 3 Department of Cell and Molecular Biology, Karolinska InstitutetStockholm, Sweden; 4 Center for Anatomy and Cell Biology, Department of Cell Biology and Ultrastructure Research, Medical University of ViennaAustria; 5 Institute of Neuroscience and School of Biology, University of Newcastle upon TyneUK; 6 Department of Neuroscience, Linné Center of Excellence in Developmental Biology and Regenerative Medicine (DBRM), Karolinska InstitutetStockholm, Sweden

**Keywords:** Bruchpilot, synapse, neuron, neurotransmission, release, visual

## Abstract

In a synaptic active zone, vesicles aggregate around a densely staining structure called the presynaptic density. We focus on its three-dimensional architecture and a major molecular component in the locust. We used electron tomography to study the presynaptic density in synapses made in the brain by identified second-order neuron of the ocelli. Here, vesicles close to the active zone are organized in two rows on either side of the presynaptic density, a level of organization not previously reported in insect central synapses. The row of vesicles that is closest to the density's base includes vesicles docked with the presynaptic membrane and thus presumably ready for release, whereas the outer row of vesicles does not include any that are docked. We show that a locust ortholog of the *Drosophila* protein Bruchpilot is localized to the presynaptic density, both in the ocellar pathway and compound eye visual neurons. An antibody recognizing the C-terminus of the Bruchpilot ortholog selectively labels filamentous extensions of the presynaptic density that reach out toward vesicles. Previous studies on Bruchpilot have focused on its role in neuromuscular junctions in *Drosophila*, and our study shows it is also a major functional component of presynaptic densities in the central nervous system of an evolutionarily distant insect. Our study thus reveals Bruchpilot executes similar functions in synapses that can sustain transmission of small graded potentials as well as those relaying large, spike-evoked signals. J. Comp. Neurol. 520:384–400, 2012. © 2011 Wiley Periodicals, Inc.

An important aim in neuroscience is to discover the structural and molecular mechanisms that underpin synaptic transmission. Of particular importance are the questions of how synaptic vesicles are recruited, prepared, and then directed to their sites of release. Vesicles assemble and are released at regions called active zones that are characterized by a number of ultrastructural specializations, including densely staining structures whose shape varies between species and, in vertebrates, between different synapses in the same animal (Zhai and Bellen,^2004^). We use the term “presynaptic density” (PD) for this structure. There is now good evidence from vertebrate neuromuscular junctions (of frogs, Harlow et al.,^2001^; mice, Nagwaney et al.,^2009^) that the PD plays a significant role in directing vesicles to their release sites.

For invertebrates, much attention has been focused on the neuromuscular junction of *Drosophila* (Kittel et al.,^2006^; Wagh et al.,^2006^; Fouquet et al.,^2009^; Jiao et al.,^2010^). But *Drosophila* neuromuscular junctions could be relatively specialized synapses: first, many neuromuscular junctions function like a relay that ensures a motor neuron spike reliably evokes a large postsynaptic response, followed by a twitch in a muscle cell. In contrast, synapses in the central nervous system normally evoke small postsynaptic potentials that are integrated with others, and some convey small, graded changes in membrane potential. Second, diptera diverged from other insect lines 330–340 million years (Gaunt and Miles,^2002^), and advanced dipteran flies such as *Drosophila* have a more elaborate PD than other arthrophods that is T-shaped rather than simple and bar-shaped in cross-section (Shaw and Meinertzhagen,^1986^). Common principles for the organization of PDs are thus not well understood. Comparative ultrastructural studies in synapses from different animals and with different physiological characteristics may aid to elucidate common functional properties of PDs.

In vertebrates, recent studies using electron tomography revealed 3D features including a polyhedral shape for PDs in brain synapses (Zampighi et al.,^2008^) and a central beam with sideways-projecting ribs in neuromuscular synapses of frogs (Harlow et al.,^2001^) and mice (Nagwaney et al.,^2009^). In contrast, synapses operating with graded potentials, such as the sensory or second-order neuron outputs of the eye and ear, contain a ribbon that reaches into the cytoplasm and to which vesicles are attached (e.g., Lenzi et al.,^1999^). Such a clear distinction in shape depending on functional characteristics has not been described in arthropods. For example, in locusts no differences have been described between the morphologies of synapses in thoracic ganglia made by spiking neurons (Watson and Burrows,^1981^) with those made by nonspiking local interneurons (thoracic ganglia, Watson and Burrows,^1988^) or by ocellar L-neurons that use graded potentials (Leitinger and Simmons,^2002^; Simmons and Littlewood, 1989).

In insects, only the protein Bruchpilot (BRP) has so far been identified as a molecular component of PDs (Fouquet et al.,^2009^). BRP is a member of the ELK/Cast family (Wagh et al.,^2006^), forms an integral part of T-bars of *Drosophila* neuromuscular junctions (Kittel et al.,^2006^; Wagh et al.,^2006^), and has also been localized to *Drosophila* photoreceptor terminals (Hamanaka and Meinertzhagen,^2010^). There is evidence that the N-terminus of BRP could play a role in clustering calcium channels at the active zone (Fouquet et al.,^2009^), whereas the C-terminus tethers vesicles to the PD (Hallermann et al.,^2010^).

We chose to study synapses made by identified neurons in the visual system of locusts because we understand the physiology and function of these neurons, and also because we can find and identify their profiles in electron microscope sections without intracellular staining. First, we studied synapses made by large, second-order neurons (L-neurons) of the ocelli, or simple eyes. These carry graded changes in membrane potential rather than action potentials to regulate neurotransmitter release (reviewed in Simmons,^2002^). Transmitter is continually released from L-neurons in darkness, requiring steady replenishment of synaptic vesicles, which contrasts with neuromuscular junctions where transmitter release is infrequent (Simmons,^2002^; Simmons and de Ruyter van Steveninck,^2005^). Second, we examined synapses made by photoreceptors with large second-order neurons in the lamina (first optic neuropil) whose repetitive nature makes it easy to identify structures (Nowel and Shelton,^1981^). They also carry graded potentials. Third, we examined synapses made in the lobula onto the lobula giant movement detectors (LGMD and LGMD2), neurons that detect rapidly approaching objects (Rind and Simmons,^1992^, ^1998^; Simmons and Rind,^1997^) and whose dendrites can be readily identified. They receive a regular array of synapses from presynaptic neurons that originate in the medulla and are likely to carry all-or-none spikes (Rind and Simmons,^1998^).

To reveal common principles in the PD architecture, we first visualized the arrangement of vesicles around the PDs of ocellar L-neuron outputs and described their 3D structure using electron tomography. We found that vesicles are arranged in a regular way, which differs from the spiking larval neuromuscular junctions in *Drosophila* previously described by Jiao et al. (2010). Second, we established the expression of a BRP ortholog in locusts with high sequence similarity to *Drosophila*. We localized the BRP ortholog to the active zones of output synapses of ocellar L-neurons, and of those made by photoreceptors with large second-order neurons, as well as those made from presynaptic neurons onto the lobula giant movement detectors. Furthermore, we present evidence from electron tomography that the BRP ortholog is an integral part of the PD of synapses in the locust visual system.

## MATERIALS AND METHODS

### Animals

Adult locusts of the species *Schistocerca gregaria* or *Locusta migratoria* were bought from either Zoo Muser in Graz or LiveFoods (Somerset, UK). They were cooled on ice before dissecting the brain in insect saline, as published previously (Leitinger et al.,^2004^). Heads of *Drosophila melanogaster* (wildtype) were obtained by freezing the animals in liquid nitrogen and sieving.

### Primary antibody characterization

Mouse monoclonal anti-BRP antibody (mAb nc82, raised against *Drosophila* head homogenate, gift of Prof. Dr. Erich Buchner, Würzburg, Germany, now in the Developmental Studies Hybridoma Bank, Iowa City, IA) was used as a primary antibody. Labeling with nc82 is absent in BRP null mutants in *Drosophila* (Kittel et al.,^2006^), and nc82 was shown to label amino acids 1,390–1,740 of BRP (Fouquet et al.,^2009^). To demonstrate that nc82 labels an antigen of an appropriate size in *S. gregaria* and *L. migratoria*, we used western blotting.

For the western blot, dissected brains of locusts and heads of adult flies were homogenized. Proteins were lysed under reducing conditions in 0.01M Tris buffer, pH 7.4, with 1% sodium dodecyl sulfate and 1% sodium orthovanadate. After 10 minutes at 70°C, protease inhibitor (Roche Complete, Roche, Mannheim, Germany) was added at 1:12.5 dilution and the lysates incubated for 5–10 minutes at 90–95°C and centrifuged for 10 minutes at 16,000*g* at 4°C and the supernatant used for electrophoresis. For electrophoresis, we used a high molecular weight detection system (Invitrogen, Lofer, Austria) consisting of 1-mm-thick NuPage Tris-Acetate gel with 3-8% gradient, (EA0375 BOX) and Tris-Acetate SDS Running buffer (LA0041) with addition of 0.25% (v/v) NuPage antioxidant (NP0005) in the upper chamber. The gels were charged with 10, 20, or 40 μg of protein lysate per lane. Samples were prepared according to the manufacturer's instructions in Sample Buffer (NP0007), with 10% Reducing Agent (NP0004, containing 500 mM dithiothreitol). Electrophoresis was made for 1 hour at room temperature at 150 V, and the blots were made using a wet system, with NuPage Transfer buffer (NP0006-1) with 0.1% NuPage antioxidant (NP0005) for 2 hours at room temperature. As a standard for the molecular weights of the proteins, we used both HiMark Prestained High Molecular Weight Protein standard and Magic Mark standard, both from Invitrogen. Immunodetection was performed using Invitrogen Western Breeze (7104 and 7106) detection systems with chemiluminescent detection according to the manufacturer's instructions using the primary antibody (nc82) at 1:250 dilution.

### Sequence comparisons

The nucleotide sequence of the BRP ortholog in migratory locusts was published by Chen et al. (2010). NCBI's basic local alignment search tool (BLAST, http://blast.ncbi.nlm.nih.gov/Blast.cgi) was used to calculate the putative amino acid sequence of the locust BRP ortholog and to compare the amino acid sequences of *Drosophila* BRP and its locust ortholog.

### Immunofluorescence

Three brains taken from adult *S. gregaria* were immunofluorescently labeled as stated previously (Leitinger et al.,^2004^). In brief, the dissected brains were fixed in a 4% formaldehyde solution in 0.1% phosphate buffer (PB), pH 7.4. They were then embedded in a gelatin solution and sectioned at 50 μm thickness using a vibratome. The vibratome sections were fluorescently labeled with nc82 as a primary antibody (diluted 1:50) and Cy3-conjugated goat antirabbit IgG (Jackson Immunoresearch Laboratories, West Grove, PA) diluted 1:100 as a secondary antibody. The sections were examined with a Leica TCS SP2 confocal laser scanning microscope, using a 10× objective, with an emission wavelength of 543 nm and detection wavelengths of 565–518 nm.

### Specimen preparation for electron microscopy

We examined both samples of brains of adult *S. gregaria* that were embedded in epoxy resin and vibratome sections of brain of adult *S. gregaria* that had previously been labeled with nc82 antibody and were then embedded in epoxy resin. For embedding, the brain samples were perfused with 2.5% paraformaldehyde, 2.5% glutaraldehyde, 0.2% picric acid, 10% sucrose in 0.1M PB, pH 7.2, for 2 hours. After being rinsed in the same PB the specimens were postfixed with 2% osmium tetraoxide, dehydrated, and embedded in TAAB epoxy resin (TAAB Laboratories, Reading UK).

### Preembedding immunogold labeling

For thin section viewing (conventional electron microscopy), the labeling was performed on vibratome sections following protocols described elsewhere (Leitinger et al.,^2004^). A brief summary of the protocol and changes are given below. The following antibodies were used on vibratome sections of the brain of *S. gregaria*: primary antibody: nc82 diluted 1:100. Secondary antibodies: 1.4 nm Nanogold-coupled goat antimouse IgG (Nanoprobes, Yaphank, NY), diluted 1:200. The antibodies were diluted in 0.1 M glycine, 0.8% bovine serum albumin, 0.1% fish gelatin, and 0.05% saponin in phosphate-buffered saline (PBS), pH 7.4. After dissecting the specimens were fixed using 0.1% glutaraldehyde, 2% paraformaldehyde, and 0.2% picric acid in 0.1 M PB, pH 7.4, for 2 hours at room temperature. Subsequently, 70-μm-thick vibratome sections were made and fixed again for 10 minutes in the same fixative, permeabilized in ethanol (10%, 30%, 50%, 30%, 10% ethanol for 2 minutes each), and then stained in a free-floating procedure. Nc82 was applied overnight at 4°C and the secondary antibody for 4 hours at room temperature. A silver enhancement step was carried out as previously described (Leitinger et al.,^2000^) using IntenSE M (cat. no. RPN491, AuroProbe, GE Healthcare, formerly Amersham, Little Chalfont, UK) with addition of one part of a 30% gum arabic solution to two parts of the silver enhancement solution for 30–40 minutes. Then the sections were rinsed in 0.15M silver nitrate solution and gold-toned to exchange the silver particles with gold particles (Laube et al.,^1996^; Leitinger et al.,^2000^). For the gold-toning reaction, 0.05% gold chloride was used diluted in 150 mM sodium acetate buffer, pH 5.6, for 7 minutes at room temperature. No background staining was detectable on thin sections when adhering to the silver enhancement and gold-toning times. These sections were then postfixed in 2% glutaraldehyde, 2% paraformaldehyde, and 0.2% picric acid in 0.1M PB, postfixed in 1% osmium tetra oxide for 30 minutes at room temperature, dehydrated in acetone, and embedded in TAAB epoxy resin.

Specimens stained using this preembedding protocol were also used for electron tomography. For the tomograms, silver enhancement time was reduced to 15 minutes in order to obtain smaller gold particles, and we used fluoronanogold goat antimouse Fab fragments (Nanoprobes), diluted 1 in 20 instead of nanogold coupled to whole IgG molecules. The fragments are considerably smaller than whole IgG molecules and thus using fragments reduces the distance between the gold granules and their epitope.

### Electron microscopy

Thin sections ≍70-nm-thick were contrasted with uranyl acetate and lead citrate and viewed with a Zeiss EM 902 transmission electron microscope. Digital images were taken using a ProScan slow scan CCD-camera at a microscope magnification 20,000×. The iTEM 5.0 Universal TEM Imaging Platform (Olympus Soft Imaging Solutions, Münster, Germany) was used to measure the distances between the gold granules. Statistics were done using Statgraphics Centurion XV software; the graphs were either made with the same software or using Sigma Plot for Windows 10.0. Images for which a tilting stage was required were made at 120 kV with an FEI Eindhoven, Netherlands) Tecnai G2 20 electron microscope at 11,500× magnification using a Gatan US1000 digital camera.

### Electron tomography of unlabeled sections

Semithin 200-nm sections were cut and stained with uranyl acetate and lead citrate. Protein A coated colloidal 10 nm gold particles (EMS, Hatfield, PA) were applied at 1:3 dilution for 10 minutes and used as fiducial markers to align images in tilt series. The grids were then washed twice in deionized water and dried. Tilt series were collected at 200 kV in an FEG CM200 FEI microscope equipped with a slow scan 2048 × 2048 TemCam-F224 CCD camera and EM-MENU 3.0 software for automated data collection (TVIPS, Gauting, Germany). The images were recorded with the CCD camera at magnification of 20,000× (pixel size at the specimen level of 7.84 Å). Tilt series were collected with one-degree increments with the aim to cover the range between −60 to +60 degrees. The collected images were displayed in the XPIX program and fiducial marker positions determined semiautomatically followed by the alignment of the images. The 3D reconstructions were computed using the weighted backprojection method (Skoglund et al.,^1998^). Low-pass noise filters were applied with a cutoff at 6 nm and the reconstructions analyzed in either BOB (http://www.3tag.com/bobicol.html) or Amira 5.3.0 software (Visage Imaging). For this, objects such as vesicles, the cell membrane, and the PD were segmented manually. Image segmentation is a procedure that aids recognition of different image features as belonging to the same object as each other, so it is useful for 3D reconstruction of objects. We thus made two 3D models from one tomogram of a locust ocellar L-neuron output synapse: first, in order to show the geometry of the synapse a model restricted to the base of the PD, the cell membrane, and the vesicles; second, in order to show ultrastructural details of the PD, another model that included filamentous connections between the vesicles, and features such as the legs that connect the PD to the cell membrane and extensions of the density toward the vesicles. Whereas most objects were segmented manually by tracing their outlines in every virtual section, the plasma membrane was traced only in every third section and its outline was then interpolated between virtual sections. The vesicles were traced by drawing their outline once in a section through the vesicle center in xy, xz, and yz planes, respectively. Their surface was then interpolated using the “wrap” function.

The ImageJ program (Image Processing and Analysis in Java, NIH, Bethesda, MD) was used to measure the distances and diameters of the vesicles as well as the length and width of the PD including the extensions of the PD in 2D. All lengths were measured using the tool “straight line selection”. The values were then transferred to a data window and were calculated using Microsoft Excel.

### Electron tomography of immunogold-labeled preparations

The 200-nm-thick sections taken from samples labeled with immunogold particles were stained using lead citrate and uranyl acetate. The tilt series were collected using a Tecnai Spirit Biotwin FEI electron microscope operating at 120 kV and with a Zeiss Libra 120 with an in-column energy filter and a Gatan Ultrascan 2k x 2k CCD camera. Tilt series were collected at 10,000, 16,000, and 18,000 magnification, tilting range −60° to +65° with images recorded digitally at 1° intervals. Once recorded, the images were aligned and reconstructed using a SIRT algorithm, which improves the signal-to-noise ratio, with the Explore 3D software package (FEI). Image segmentation of one reconstruction of an active zone was performed using Amira software. The segmented objects included eight gold granules, the vesicles, the cell membrane, and the PD. This reconstruction allowed drawing finer details of the PD than the electron tomograms of unlabeled sections.

### Figures

The brightness and contrast of the figures were adjusted and rescaled to 300 dpi resolution.

## RESULTS

### Organization of vesicles around the PD at synapses made by ocellar L-neurons

**Figure 1:**
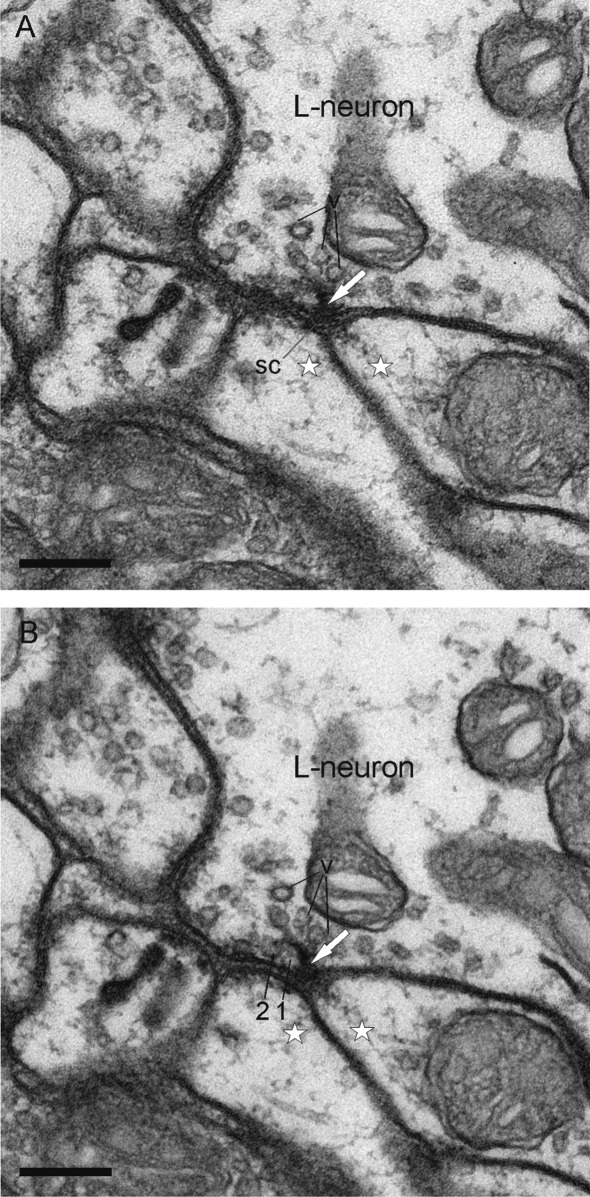
Conventional electron microscopy of a locust ocellar L-neuron output synapse. The active zone is characterized by a presynaptic density (arrows) around which vesicles (v) cluster. **A**: Stage tilted to −10 degrees to reveal the synaptic cleft (sc). **B**: Stage tilted to +10 degrees to reveal the vesicles close to both the cell membrane and the presynaptic density. The vesicles are labeled with 1 and 2. Asterisks indicate postsynaptic neurites. Scale bars = 200 nm.

**Figure 2:**
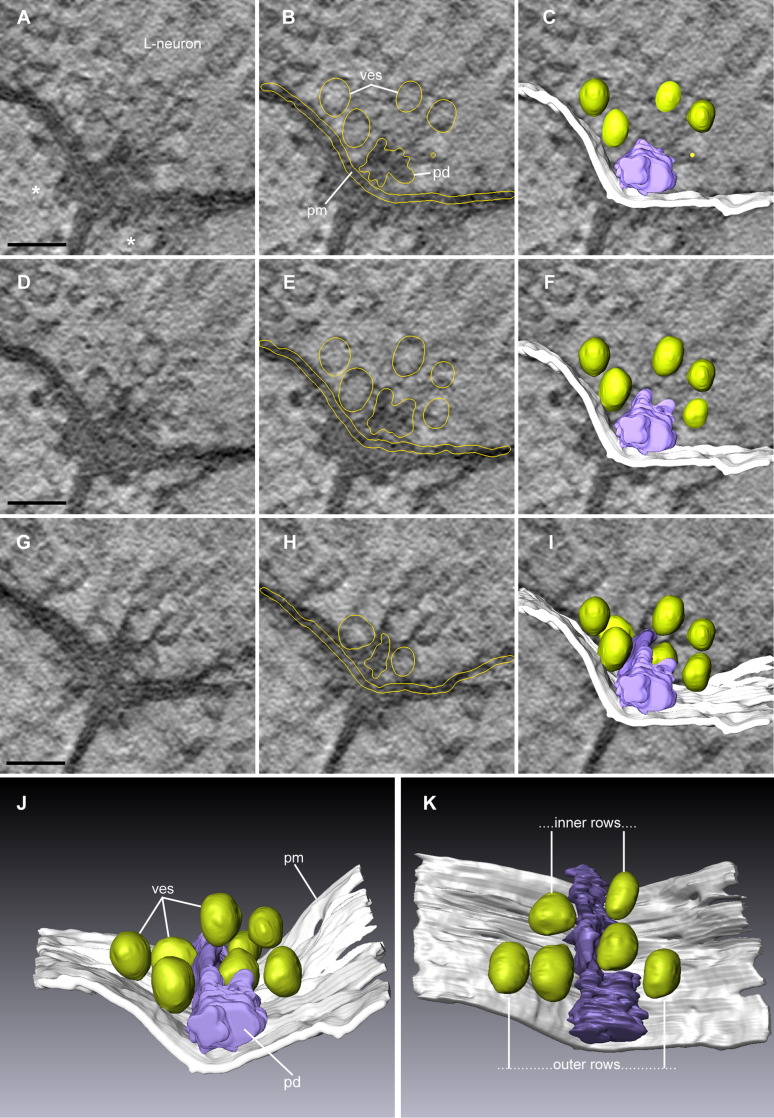
Geometry of ocellar L-neuron output synapses as revealed in electron tomograms. **A,D,G**: Sequence of virtual images made from an electron tomogram of an L-neuron's output synapse in three different planes of section. **B,E,H**: The same virtual images with the contours of the objects that were segmented highlighted in orange. **C,F,I**: The same virtual images with the segmented 3D model shown as an overlay. **J**: Side view of the 3D model showing that the vesicles are arranged around the presynaptic density, forming a corona in cross-section. **K**: The same model as in J in top view showing only those vesicles that are associated with the plasma membrane. These are arranged in two inner and two outer rows either side of the presynaptic density. pm, plasma membrane; pd, presynaptic density; ves, vesicles. Color code: green, vesicles; purple, presynaptic density; white, plasma membrane. Scale bars = 80 nm.

**Figure 3:**
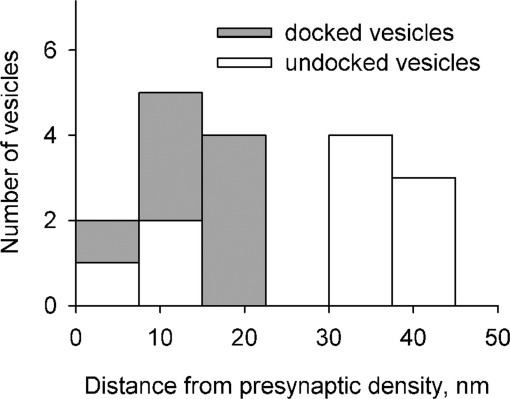
Histogram of the distribution of distances between docked and undocked vesicles and the PDs. The docked vesicles are all situated in an inner row, close to the presynaptic bar, whereas the undocked vesicles are mostly (but not only) situated in an outer row. A bin width of 7.5 nm was chosen to represent the two rows of vesicles optimally. Three tomograms taken from different synapses were analyzed. *n* = 10 for docked vesicles and 11 for undocked vesicles.

Conventional electron micrographs of synapses made from ocellar L-neuron axons show that a corona of vesicles encircles each PD (Fig. 1A,B). Serial section electron microscopy has previously shown that each connection consists of hundreds or thousands of active zones, each with an aggregation of vesicles around a PD (Simmons and Littlewood,^1989^; Littlewood and Simmons,^1992^; Leitinger and Simmons,^2002^). The active zones are elongated and vary in length from less than 70 nm to 1.5 μm (Leitinger and Simmons,^2002^). Using a tilting stage enabled us to visualize the synaptic cleft between the membranes of the pre- and postsynaptic neurites (Fig. 1A), or vesicles that are close to both the cell membrane and the PD (labeled with numbers 1 and 2, respectively, in Fig. 1B). For a detailed reconstruction of the vesicle arrangement around the PD, we made 200-nm-thick cross-sections of PDs. By examining the electron tomograms of five cross-sections taken from different synapses (Fig. 2A–J), we can now reveal a high degree of order in the arrangement of vesicles next to the PDs. This order was not visible when using conventional electron microscopy (Leitinger and Simmons,^2002^), which gives no representation of the 3D structure within a single section. The new feature that the electron tomograms of the active zones show is that vesicles are arranged in a very regular manner near the base of the PD—the edge of the PD that is closest to the plasma membrane (Fig. 2K). We focused on vesicles that were within 50 nm of the base of the PD and whose edge was less than a vesicle width from the presynaptic membrane. Vesicle width in electron tomograms was 34 ± 9.85 nm (mean ± SD; *n* = 34) and all of these vesicles were attached to the PD or to other vesicles by filamentous strands of electron-dense material. We distinguished two distinct rows of vesicles with respect to both the plasma membrane and the PD when viewed through the vesicle center: within 10 nm of the PD base, and about 30 nm from its base. We also determined whether these vesicles appeared docked (in direct contact with the plasma membrane) or undocked (the space between vesicle membrane and plasma membrane being bridged with macromolecules of 6.02 to 21.92 nm length, median value: 10.25; *n* = 42). When we followed tomograms of three PDs through the thickness of the section (a total length of about 600 nm along the active zone) we found nearest the PD 11 vesicles that appeared both docked at the plasma membrane and another three that were not docked; and about 30 nm away from the base we found seven vesicles, none of which were docked. The median distance between the membranes of the docked vesicles and the PDs was 9.64 nm (range 7.07–21.62 nm), whereas the median distance between the membranes of the undocked vesicles and the PDs was 33.55 nm (range 6.37–43.57 nm) (Fig. 3). A Wilcoxon test showed that the distance between the PD and docked vesicles differed significantly (*P* < 0.05) from the distance between the PD and undocked vesicles.

### Organization of branched extensions of the PD

**Figure 4:**
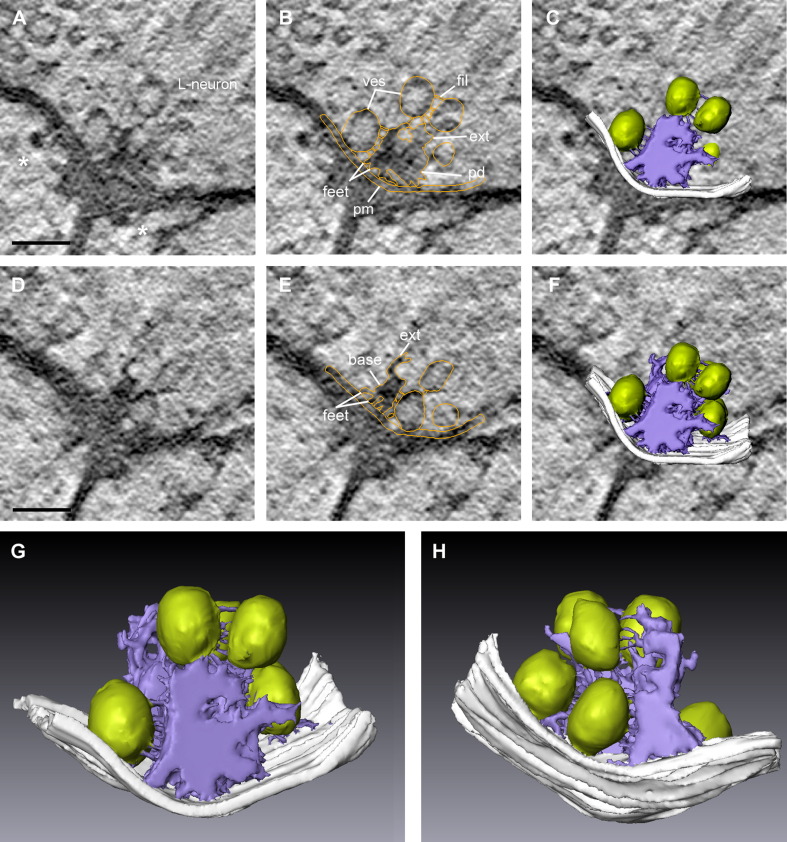
Electron tomogram showing ultrastructural details of the same presynaptic density (pd) as in Figure 2. The presynaptic density consists of a base, it is connected to the plasma membrane (pm) with legs and has extensions (ext) that reach into the cytoplasm. Thin filamentous material (fil) connect the vesicles with each other and with the presynaptic density. **A,D**: Sequence of virtual images made from an electron tomogram of an L-neuron's output synapse in two different planes of section. **B,E**: The same virtual images as in A,D with the contours of the objects that were segmented highlighted in orange. **C,F**: The same virtual images with the segmented 3D model shown as an overlay. Color code: green, vesicles; purple, presynaptic density and filamentous connections; white, plasma membrane. Scale bars = 80 nm.

We used image segmentation to identify the fine structural features of the PDs and their components (Fig. 4A–F). We found that a PD has an electron-dense base, connected to the cell membrane by leg-like structures (Fig. 4G,H) in a similar way to the “pegs” in frog and mouse neuromuscular junctions (Harlow et al.,^2001^; Nagwaney et al.,^2009^). Such structures were also observed by Fouquet et al. (2009) and Jiao et al. (2010) in *Drosophila* neuromuscular junctions. The electron tomograms allowed us to resolve for the first time in locust PDs another striking feature, which was that structures a little like squat antlers extend into the cytoplasm (Fig. 4G,H) at certain intervals from a PD toward vesicles. These squat structures may be homologous with the more extended structures located on PDs at fly synapses (Jiao et al.,^2010^). Filamentous proteins also appeared to surround the membranes of the synaptic vesicles and formed connections from vesicles to other vesicles, to the base of the PD or its extensions, or to the presynaptic membrane (Fig. 4G,H). Thus, filamentous proteins appeared to connect the vesicles with each other into chains that could reach to the plasma membrane distal from the PD. The measured dimensions of these features are summarized in Table 1.

**Table 1:** Proportions of the Components of a Presynaptic Density (pd) of a Locust Ocellar L-neuron Output Synapse, Measured After Segmenting the pd and Its Components

	Mean value (nm)	Standard deviation (nm)	*n*
Distance between base of the pd and plasma membrane	19.09	7.54	77
Height of base of pd	29.54	14.19	22
Width of base of pd	38.03	8.12	26
Length of connecting filaments between vesicles	15.78	4.68	16
Length of extensions– distal part	32.49	9.32	41
Length of extensions– proximal part	20.22	4.46	14

### Monoclonal antibody nc82 recognizes proteins of similar molecular weight in *Drosophila* and locusts

**Figure 5:**
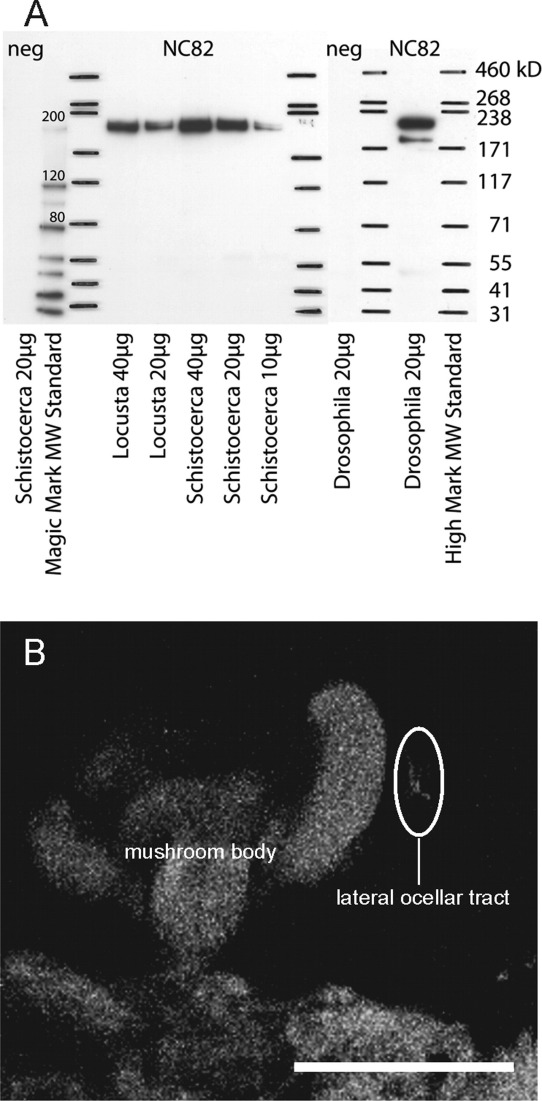
The antibody nc82 recognizes proteins of similar molecular weight in *Drosophila* and locusts. **A**: Nc82 labeling in a western blot of brain homogenate of locusts and a head homogenate of *Drosophila*. Different amounts of protein from two different locust species (*Schistocerca gregaria, Locusta migratoria*) were blotted. In both locust species and in *Drosophila*, nc82 labels a double band. Note that the two molecular weight standards give slightly different results (Magic Mark being lower than High Mark). neg, Negative control. **B**: Overview of nc82 immunofluorescence in the lateral protocerebrum of a locust. Labeling is confined to the synaptic neuropil areas (e.g., mushroom body and lateral ocellar tract). Scale bar = 250 μm.

Our further investigation focused on the molecular composition of the PD. Since the BRP protein is a known integral part of PDs (T-bars) in *Drosophila* neuromuscular junctions (Fouquet et al.,^2009^), we examined its expression and fine structural location in locust visual synapses. First, to determine whether the BRP protein is conserved between evolutionarily distant insect species, we calculated the putative amino acid sequence of the locust BRP ortholog and found 34% of the amino acids were matching between the *Drosophila* and locust in the total protein. We then probed western blots of locust and fruit fly tissue with nc82, an antibody directed against BRP in fruit flies (Wagh et al.,^2006^; Fouquet et al.,^2009^). In brain homogenates of both locust species *S. gregaria* and *L. migratoria*, this antibody detected a band doublet around ≍220 kD (Fig. 5A). The fact that these bands are of a similar molecular weight to the two bands that the antibody recognizes in *Drosophila* (Fig. 5A; Wagh et al.,^2006^) suggests a high degree of conservation of the BRP molecule between insect species. The epitope for nc82 lies between C-terminal amino acids 1,390–1,740 of the 1,740 amino acid long molecule in *Drosophila* (Fouquet et al.,^2009^) and, because the antibody binds to it in such distinct genera, has apparently been highly conserved. In fact, an NICBH blast shows a region of high homology between amino acids 1,284–1,728, closely overlapping with the putative binding site of nc82. Our blots showed the *Drosophila* band doublet at 190 and 200 kD, which compares well with 170 and 190 kD observed by Wagh et al. (2006). Note that our own results differ by ≍12% when comparing the molecular weights indicated by a Magic Marc standard with those indicated by a High Molecular Weight standard (compare the second with the third lane in Fig. 5A). Immunofluorescence of nc82 in the lateral protocerebrum shows that staining is confined to the synaptic neuropil areas, e.g., within the mushroom body or the lateral ocellar tracts (Fig. 5B).

### Thin sections labeled with nc82 reveal the BRP ortholog distribution at the active zones of identified visual synapses

**Figure 6:**
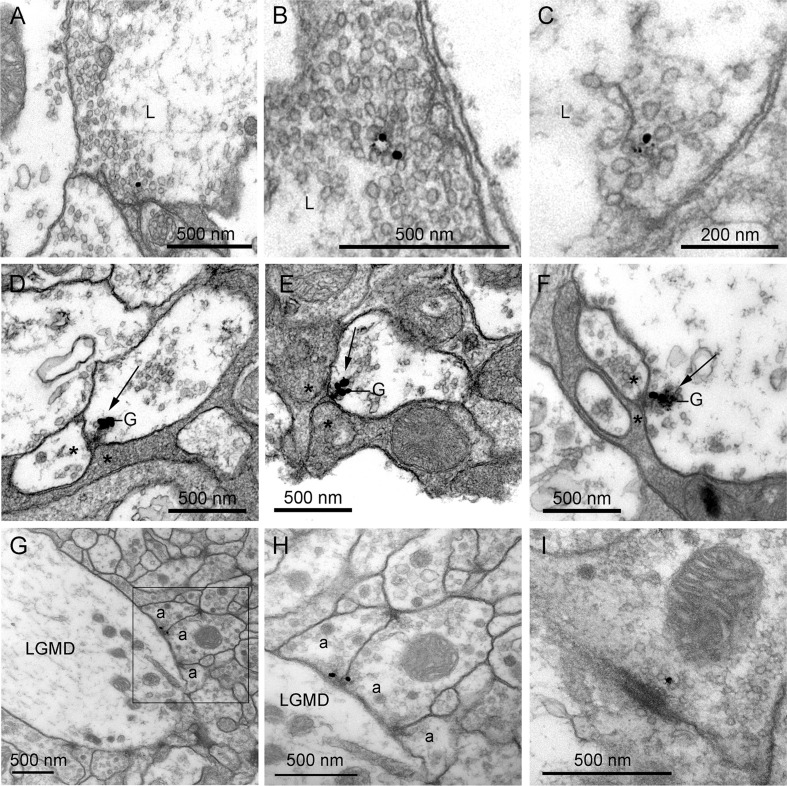
A BRP ortholog can be localized to the active zone of output synapses of several identified visual neurons in locust. EM immunogold labeling of BRP using nc82 visualized on thin sections. **A–C**: Output synapses of ocellar L-neurons (L) in the lateral ocellar tract. Note that the gold granules are close to filamentous electron-dense material in C, which is an oblique section. **D–F**: Unidentified neurites in the lamina neuropil. The gold particles (G) are precisely localized along the tip and sides of the PDs (arrows). The base of the PDs remains unstained. Asterisks indicate postsynaptic neurites. **G–I**: Electron micrographs of the lobula complex showing BRP staining in neurons that make synapses onto the LGMD1 or LGMD2 (labeled with a in G,H). I: A longitudinal section of a labeled synapse.

To characterize the association between the BRP ortholog and the PDs further, we examined closely the distribution of BRP using conventional electron microscopic immunocytochemistry with the antibody nc82. We examined sections of the ocellar tracts and found that the active zones of output connections of ocellar L-neurons clearly labeled with immunogold granules (Fig. 6A–C), which, in oblique sections, appeared to be close to filamentous material associated with the vesicles (Fig. 6C). To test whether other identifiable neurons also expressed the BRP ortholog, we labeled BRP in two other visual neuropil areas that each contain readily identifiable neurites: first, in the lamina (first optic neuropil) whose repetitive nature makes it easy to identify structures (Nowel and Shelton,^1981^); and second, in the lobula complex (third optic neuropil), where we were able to identify synapses onto unique branches of the LGMD and LGMD2 neurons because their neurites are the widest in the lobula. In the lamina, the gold granules were consistently located at active zones of synapses decorating the PDs in a variable density (Fig. 6D–F). Many synapses of unidentified or identified neurons were labeled, including the output synapses of photoreceptor cells (data not shown). In the lobula, synaptic active zones, including those involving dendrites of the LGMD and LGMD2 as postsynaptic elements, clearly exhibited staining in both cross and longitudinal sections (Fig. 6G–I).

**Figure 7:**
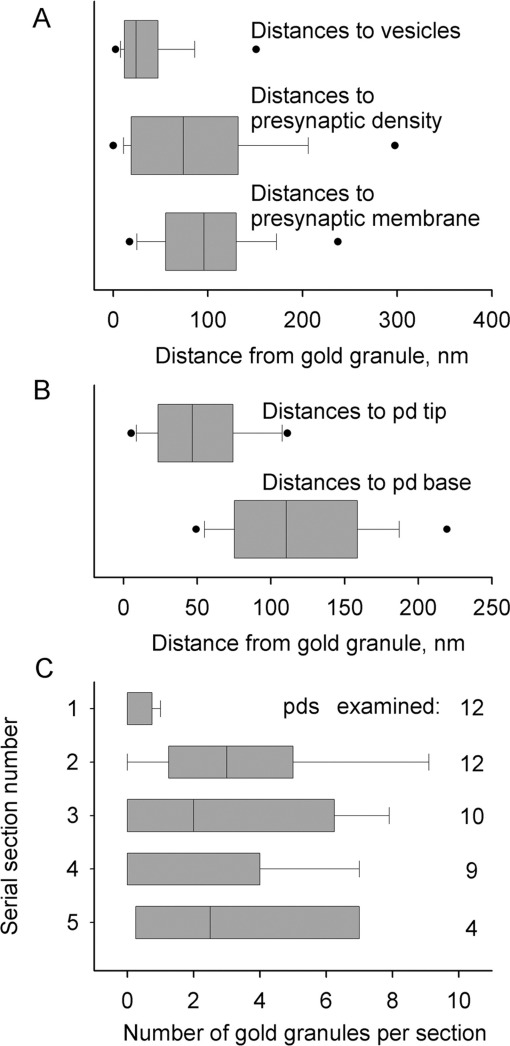
Statistical data as box-and-whisker plots of the location of BRP immunogold granules, labeled with nc82 primary antibody, show that the gold granules label a structure associated with the presynaptic density (pd) at a visible distance to the cell membrane. **A**: Distances between the gold granules in the lamina and the vesicles, the presynaptic density, and the cell membrane. The gold granules were closest to the vesicles, followed by the presynaptic density and the membrane. The distances between gold granules and presynaptic density were statistically significantly lower than the distance between gold granules and cell membrane. Gold granules were randomly selected. *n* = 60. **B**: Distances of gold granules to the tip of the presynaptic bar compared to their distance to the base of the bar. The gold granules are closer to the tip than to the base. *n* = 42. **C**: BRP is an integral component of the active zone material. Numbers of gold granules as markers for BRP associated with the active zones of unidentified visual synapses in serial section electron microscopy. Twelve PDs were followed on serial sections and the number of gold granules associated with them counted. The counts were then arranged so that the border section was always counted as section 1 (so section 0 was always a section on which the presynaptic density was not yet visible). In B,C, 5th and 95th percentile outliers are shown.

The gold granules were separated from the cell membrane. To describe their location in more detail, we measured distances between randomly selected gold granules and their nearest clearly visible structural feature such as the border of a PD, a synaptic vesicle, or the cell membrane. In a survey of 60 gold granules, vesicles were the closest features (75% of granules), followed by PDs (22% of granules) and cell membrane (just 3% of granules). Median values of measurements of the distances of each gold granule to the nearest vesicle, the nearest PD, and the nearest cell membrane showed that the vesicles appeared closest to the gold granules on thin sections, followed by the PDs and the cell membranes. The distances between granules and vesicles were significantly lower than the distances between granules and cell membranes (Fig. 7A; Mann–Whitney test; *P* ≤ 0.001); the distance between granules and PD did not differ significantly from the other distances.

To obtain more data on the distribution of gold granules near PDs, we marked gold granules that were situated within 100 nm of the PD border and thus could clearly be associated with a particular PD cut in cross-section, and we measured the distances between each granule and both the base of a density and the tip of the density. For 95% of granules, the tip of the density was closer than its base (*n* = 42). A box-and-whisker plot (Fig. 7B) of the distances between the gold granules and the tip and base of the PDs associated with them shows that most gold granules were between 25 and 60 nm from the tip of the PD, whereas they were much further (135–170 nm) away from the base of the PD. The two groups differed significantly from each other (Mann–Whitney test; *P* ≤ 0.001; *n* = 42).

### Serial sections reveal the distribution of the BRP ortholog at the PDs

To visualize the distribution of gold granules along the length of the PDs, we followed 12 different synapses on series of 3–7 sections to count the number of gold granules associated with their PD. In a consecutive series of sections through each density, we labeled the first section in which the PD was apparent as number 1, so the preceding section was number 0. The number of gold granules associated with the densities varied from section to section, ranging from 0 to 10 (48 sections counted, mean 2.17 granules, SD = 2.86). All these granules were within 50 nm of a PD; we did not find any free within the cytoplasm.

When the granule counts for different PDs were combined into a single plot, it was clear that the sections on which PDs first appear contained only one or no gold granules, whereas many more gold granules were associated with the PDs further along their length (Fig. 7C), so there was much more labeling when the PD extended through the thickness of a section than when it started within a section, signifying that nc82 labeled a structure associated with the PD. A Mann–Whitney test showed that the number of gold granules on the first sections of the synapses differed significantly from the second and third sections (asterisks in Fig. 7C; first vs. second section: *P* = 0.001; first vs. third section: *P* = 0.024). The fourth and fifth sections did not differ significantly from the first.

### On electron tomograms, the C-terminal end of BRP can be localized to extensions of the PD

Thin sections thus showed that the C-terminal part of BRP surrounded the PDs close to the synaptic vesicles and at 110.4 nm median distance to the cell membrane (*n* = 42). In order to investigate which component of the PD included BRP, we made electron tomograms of sections that had been labeled with nc82-immunogold. These were of visual synapses of unknown type from within the lamina. Two ways of examining four different tomograms showed a clear association of the gold granules with the extensions that link the base of the PD with the thin filamentous material connecting to the vesicles.

**Figure 8:**
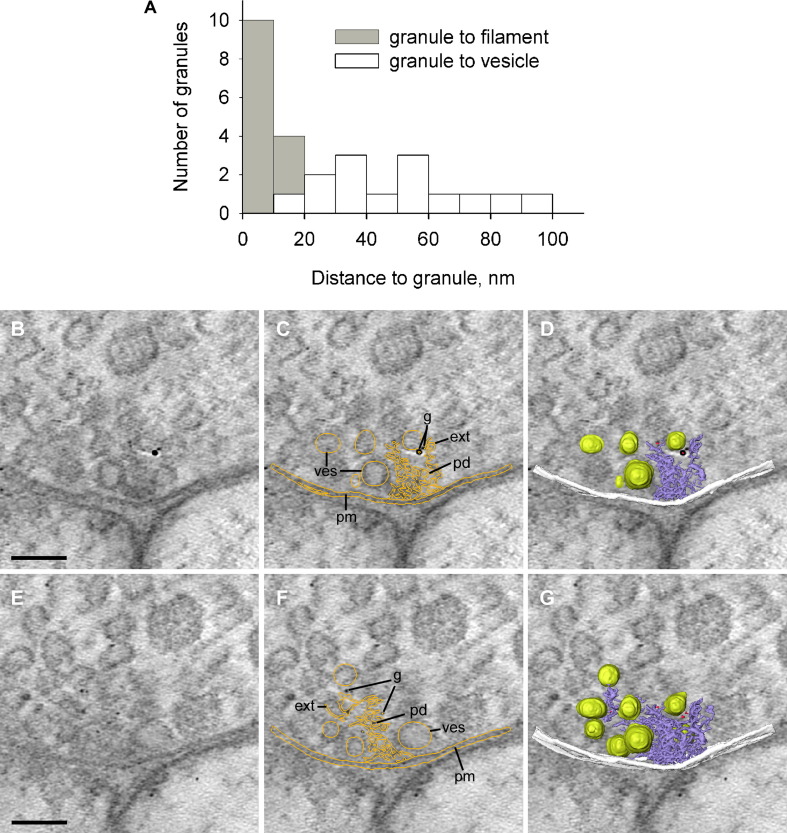
nc82 immunocytochemistry combined with electron tomography shows that extensions of the presynaptic density are labeled with nc82. **A**: The nearest neighbors of gold granules are always filamentous proteins rather than vesicles. Distribution histograms of the distances between gold granules and the nearest filamentous proteins (either extensions of the presynaptic density or filamentous material surrounding the vesicles) and vesicles as seen in electron tomograms. We did not distinguish between the extensions of the presynaptic density and the filamentous material surrounding the vesicles for this figure. Bin size 10 nm (*n* = 12). **B–G**: Image segmentation to visualize eight gold granules on one presynaptic density and their surrounding material. B,E: Sequence of virtual images made from an electron tomogram of a visual synapse of unknown type, labeled with nc82-immunogold, in two different planes of section. C,F: The same virtual images with the contours of the objects that were segmented highlighted in orange. D,G: The same virtual images with the segmented 3D model shown as an overlay. Note that the filamentous material surrounding the vesicles was omitted for reasons of clarity. g, gold granules; pd, presynaptic density; ext, extensions of the presynaptic density; ves, vesicles; pm, plasma membrane. Color code: red, gold granules; green, vesicles; purple, presynaptic density; white, plasma membrane. Scale bars = 80 nm.

First, a statistical evaluation of the distances between the borders of the gold granules and their nearest structural feature showed that filamentous material (either the filamentous material surrounding the vesicles or the extensions of the PDs) was closer to the gold granules than the vesicles (8.4 ± 3.2 nm vs. 50.2 ± 23.6 nm, means ± SD, *n* = 14; Fig. 8A). The differences between the distances were statistically significant (*P* < 0.001; Mann–Whitney *U* Rank Sum Test). The distance of about 8 nm between the gold granules and the filamentous material was to be expected bearing in mind that a primary antibody and an Fab fragment fill the space between the center of the gold granule and the material they labeled.

**Figure 9:**
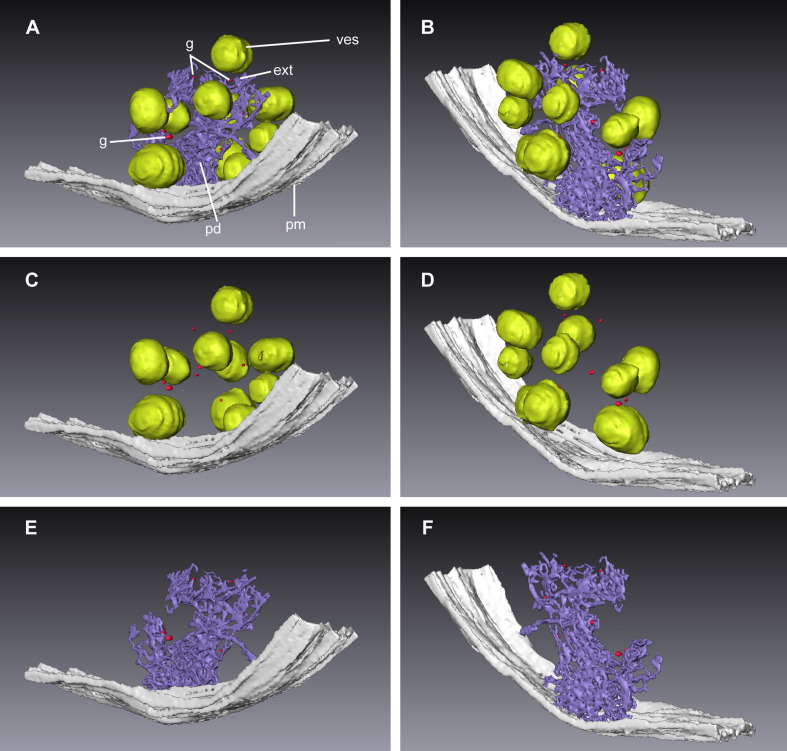
Different views of the 3D model of the immunolabeled synapse also shown in Figure 8. **A,B**: Front and back view of the synapse showing all the reconstructed features. **C,D**: Front and back view of the same synapse with the presynaptic density omitted. **E,F**: Front and back view with the vesicles omitted. g, gold granules; pd, presynaptic density; ext, extensions of the presynaptic density; ves, vesicles; pm, plasma membrane. Color code: red, gold granules; green, vesicles; purple, presynaptic density; white, plasma membrane.

Second, a 3D reconstruction showed that the extensions of the PD were labeled with gold (Figs. 8B–G, 9). We showed this in a separate tomogram from that used in Figure 8A, and we used image segmentation to reveal the following as discrete objects: gold granules; the PD, its extensions and legs; the vesicles; and the cell membrane itself (Fig. 8 B–G). The position of eight gold granules with respect to a single PD is thus shown in a model (Fig. 9A,B). Removing either the PD (Fig. 9C,D) or the vesicles (Fig. 9E,F) from the model makes it clear that, although most gold granules are associated with the vesicles, they are all more closely associated with extensions of the PD.

## DISCUSSION

We found that the synaptic vesicles are regularly arranged at the cell membrane of active zones of excitatory output synapses of locust ocellar L-neurons and we show that BRP is ubiquitous, being found in synapses that can either sustain transmission for a long time or those that transmit spike evoked signals.

### PD structure and its association with vesicles

Reconstructions of large ocellar L-neuron output synapses made from electron tomograms enabled us to examine the arrangement of vesicles around PDs in serial transverse sections along their lengths. We consistently found two rows of vesicles attached to the density and associated with the cell membrane, a level of regularity in synaptic organization at a central insect synapse that has not been previously reported. One row of vesicles, at ≍10 nm distance from the PD center, mostly (≍80%) appeared docked with the presynaptic membrane, while members of the other row, ≍30 nm from the PD, were never docked. It is very likely that the first row is the site of release, similar to the rows of docked vesicles seen in electron tomograms of frog and mouse neuromuscular junctions (Harlow et al.,^2001^; Nagwaney et al.,^2009^) in which their number and spacing correspond to fusion sites seen in quick-frozen, deep-etched material of rat or lizard neuromuscular junctions (Ellisman et al.,^1976^; Walrond and Reese,^1985^). Rather than rows, triads of vesicles were found tethered to the plasma membrane underneath the PDs (T-bars) of larval neuromuscular junctions of *Drosophila* (Jiao et al.,^2010^). Vesicles in these triads were reported to lose their direct connections with other vesicles (Jiao et al.,^2010^).

The synapses made by L-neurons can release at high rates, and their release is regulated by graded changes in membrane potential rather than spikes (e.g., Simmons and de Ruyter van Steveninck,^2005^), so they operate in a similar way to the ribbon type visual synapses in the vertebrate retina (reviewed by LoGiudice and Matthews,^2009^). Either side of the PD, over a length of ≍600 nm, we found 14 vesicles in the inner row. Given a vesicle width of 35 nm, these 14 vesicles took up 40% of the available length, which would leave some space for additional vesicles to dock. Counting only the docked vesicles (11 of the 14 in the inner row), we estimate that each 1-μm length of PD can dock 18 vesicles, which is lower than the docking capacity of ribbon synapses in cat's cone cells (60 per μm, calculated from Sterling and Matthews,^2005^). The relatively low value of our estimate may be because electron tomography allowed us to distinguish more precisely between morphologically docked and undocked vesicles. The light adaptation of the animal, its temperature, and the type of fixation may also influence vesicle packing density. The connection of L-neurons L1-3 with one identified synaptic target, DNI, is estimated to consist of up to 10,000 discrete active zones, each with its own PD of variable length (Simmons and Littlewood,^1989^). Using data from these studies, we estimate that in the ensemble of individual active zones between an L-neuron and DNI up to 52,000 vesicles could dock. This contrasts with 600 docked vesicles in the total terminal length of a cat's cone (Sterling and Matthews,^2005^), but several cones may synapse onto one interneuron. Also, a cone's synaptic ribbon is a plate-like structure that can tie a significantly larger number of vesicles because it reaches much further into the cytoplasm than an insect's PD. In vertebrates, synaptic ribbons are thought to be specialized for rapid restocking of docked vesicles, or synchronous fusion of all the vesicles held by the ribbon with the cell membrane (Matthews and Sterling,^2008^), but no differences in structure have been found in arthropods between synapses that transmit graded potentials and synapses activated by spikes

Our electron tomography of locust central synapses revealed that fine, macromolecular structures of the PD form extensions that reach out from its base into the cytoplasm, and that the base is connected to the plasma membrane with multiple peg-like structures. Strikingly, both these elements have also been revealed at PDs (T-bars) of *Drosophila* neuromuscular junctions when the tissue was high-pressure-frozen and freeze-substituted (Fouquet et al.,^2009^; Jiao et al.,^2010^). Furthermore, we found that filamentous material interlink vesicles and connect them to the PD. All the elements we found (the connections to the cell membrane, the PD and its extensions, the filamentous material) have counterparts in neuromuscular junctions of frogs and mice, termed “pegs” for the connections to the base, “beams” and “ribs” for the PD and its extensions (Harlow et al.,^2001^; Nagwaney et al.,^2009^). Because of the spacing and size of the “pegs” in vertebrates they are likely to be the locations of the membrane calcium channels (Harlow et al.,^2001^). In *Drosophila* neuromuscular junctions, vesicles tethered to the presynaptic membrane were found to be situated within 50 nm of calcium channels so that a local increase in calcium concentration would trigger their release (Jiao et al.,^2010^).

Our finding in the tomograms that fine, filamentous structures surround the vesicles needs to be interpreted with some caution because spatial resolution is not isotropic and so might be insufficient to resolve fine structures in every spatial axis. However, filamentous material interconnecting vesicles has also been found in other presynaptic zones, being described as “filaments” in rat hippocampal synapses (Siksou et al.,^2007^) and in frog saccular hair cells (Lenzi et al.,^1999^); as “spokes” in the rat neocortex (Zampighi et al.,^2008^); and as “connectors” in rat cerebrocortex or hippocampus (Fernandez-Busnadiego et al.,^2010^). Although the size and location of the filaments in rat hippocampus is consistent with synapsin as a component (Hirokawa et al.,^1989^; Siksou et al.,^2007^), synapsin knockout mice are not completely void of filaments, suggesting that there are a number of different filamentous molecules associated with vesicles (Siksou et al.,^2007^). The filaments are rearranged upon stimulation, so they may play a role in vesicle mobilization (Fernandez-Busnadiego et al.,^2010^).

### BRP as a molecular constituent of PDs in insect synapses

Many proteins are known to be associated with the active zone of synapses in vertebrates (Schoch and Gundelfinger,^2006^; Zanazzi and Matthews,^2009^), and some of these have been localized to the PD. Two proteins have so far been localized to T-bars in larval *Drosophila* neuromuscular junctions SRPK79D and BRP (Fouquet et al.,^2009^; Johnson et al.,^2009^; Nieratschker et al.,^2009^). BRP is a large structural component of the T-bars (Fouquet et al.,^2009^) and has also recently been localized to the T-bar of photoreceptor terminals in *Drosophila* (Hamanaka and Meinerzthagen, 2010). Our data from locusts demonstrates that orthoptera possess an ortholog to BRP. Using western blots and immunofluorescence, we show that the monoclonal antibody, nc82, directed against an epitope at the C-terminal part of *Drosophila* BRP, is able to recognize a band doublet in locusts, with a similar molecular weight to that recognized in *Drosophila* (Wagh et al.,^2006^). The region containing the epitope for nc82 shows high homology between locusts and *Drosophila*, and furthermore, we have found the BRP ortholog in the central nervous system of locusts in presynaptic terminals that have different, contrasting physiological characteristics. These include outputs from L-neurons and from photoreceptors, which can sustain transmitter release and can transmit small graded potentials, and those made by small-axon neurons onto LGMD dendrites, where the presynaptic neuron is likely to generate all-or-none spikes. The presynaptic neurons at these synapses use a variety of neurotransmitters: insect photoreceptors release histamine (Hardie,^1987^; Simmons and Hardie,^1988^; Gebhardt and Homberg,^2004^), whereas acetylcholine is probably the neurotransmitter of L-neurons (Leitinger and Simmons,^2000^) and of the synaptic inputs onto the LGMDs (Rind and Leitinger,^2000^; Peron et al.,^2009^). Our study significantly extends current knowledge of the distribution of the BRP protein that previously focused on *Drosophila* neuromuscular junctions (Kittel et al.,^2006^; Wagh et al.,^2006^; Fouquet et al.,^2009^), which are glutamatergic (Fouquet et al.,^2009^). Our findings indicate that BRP has a similar molecular structure and location in orthopterans and dipterans. Data from a molecular clock show that the orthopterans and dipterans separated from a common ancestor ≍350 million years ago (Gaunt and Miles,^2002^). Due to this high conservation, the antibody nc82 presents a universal tool for marking neuropil areas in several insect orders, not only in *Drosophila*, where it is widely used, as has previously been summarized by Wagh et al. (2006).

Chen et al. (2008) pioneered the use of immunogold labeling for electron tomography as a way of visualizing the proteins antibodies attached to. We found that nc82 labels the extensions of the PDs in all the visual synapses we focused on. In *Drosophila*, the BRP is probably long and thin because its C-terminal part was found at the distal ends of the T-bars, whereas the N-terminal part was found clustered with other such N-terminals at the T-bar pedestal (Fouquet et al.,^2009^). This localization for BRP corresponds well with our finding of filamentous structures in the vicinity of the vesicles in L-neuron output synapses. The pedestal of *Drosophila* PDs probably corresponds with the “pegs” discovered by Harlow et al. (2001) in vertebrate neuromuscular junctions, which closely overlap calcium channel clusters. Thus, the N-terminal part of BRP is well placed to interact with Ca^2+^-channels, which explains why characteristics of *Drosophila* BRP mutants include misplaced calcium channels, reduced evoked vesicle release, and altered short term plasticity (Fouquet et al.,^2009^). The structure of the C-terminal part of BRP resembles multifunctional cytoskeletal proteins, but shows no homology to vertebrate or *C. elegans* synaptic proteins (Wagh et al.,^2006^). The C-terminal amino acids 1,226 to 1,390 have been shown to be required for T-bar formation in *Drosophila* (Fouquet et al.,^2009^). The final 17 amino acids in the C-terminal are required to tether synaptic vesicles to the T-bar, and their loss impairs high-frequency sustained vesicle release and recovery (Hallermann et al.,^2010^). Corroborating these results, we localize the C-terminus to extensions of the PD that reach out toward vesicles in all the locust central synapses that we examined. BRP could thus be an integral part of the PD, involved in vesicle tethering in synapses that operate in many different ways, with and without spikes, with continuous or discontinuous release, not only in *Drosophila* but in other insects as well.

**Figure 10:**
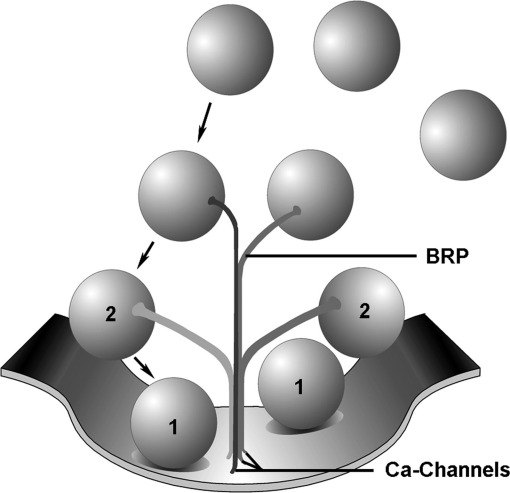
Schematic model showing how BRP molecules could bridge the space between the membrane calcium channels and the synaptic vesicles and guide the movement of vesicles from a reserve pool toward the cell membrane. Arrows indicate movement of a vesicle toward the docked location. The vesicles situated in the inner and outer rows near the cell membrane are labeled with 1 and 2, respectively. The vesicles labeled with 1 have lost their attachment with BRP and are docked with the presynaptic membrane. For clarity, only one BRP molecule per vesicle is depicted and short filaments interlinking the vesicles have been omitted from the drawing.

A scheme for the way BRP could be involved in guiding vesicles to their release sites is drawn in Figure 10, and is compatible with our results. BRP is drawn as an elongated, filamentous molecule that can bend. Other structural proteins in the PD are not drawn. When a vesicle in the reserve pool attaches to the C-terminal of a BRP molecule, the vesicle is effectively tethered to the PD. The BRP molecule then ensures that the vesicle will arrive at the correct location for docking with the presynaptic membrane, near to calcium channels. Because immunolabeling for BRP is scarce at the docking sites near the PD's base, it is likely that a vesicle detaches from BRP during or before docking.
